# Protocol of a randomized controlled trial of the fear of recurrence therapy (FORT) intervention for women with breast or gynecological cancer

**DOI:** 10.1186/s12885-016-2326-x

**Published:** 2016-04-25

**Authors:** Christine Maheu, Sophie Lebel, Christine Courbasson, Monique Lefebvre, Mina Singh, Lori J. Bernstein, Linda Muraca, Aronela Benea, Lynne Jolicoeur, Cheryl Harris, Agnihotram V. Ramanakumar, Sarah Ferguson, Souraya Sidani

**Affiliations:** Ingram School of Nursing, McGill University, Montreal, Quebec J7V 0E2 Canada; Cancer Survivorship Program, Princess Margaret Cancer Centre, University Health Network, Toronto, Ontario M5G 2C4 Canada; School of Psychology, University of Ottawa, Ottawa, Ontario K1N 6N5 Canada; Centre for Addition and Mental Health, CB, DB Therapy & H Therapy Centre, Toronto, Ontario M4T 1Z2 Canada; Department of Psychology and Psychosocial Oncology Program, The Ottawa Hospital Cancer Centre, Ottawa, Ontario K1H 8L6 Canada; School of Nursing, York University, Toronto, Ontario M3J 1P3 Canada; Auxiliary Breast Health Program, Joseph and Wolf Lebovic Health Complex, Mount Sinai Hospital, Toronto, Ontario M5G 1X5 Canada; After Cancer Treatment Transition Clinic, Women’s College Hospital, Toronto, Ontario M5S 1B2 Canada; Integrated Cancer Program, The Ottawa Hospital, Ottawa, Ontario K1H 8L6 Canada; Division of Cancer Epidemiology, McGill University, Montreal, Quebec H2W 1S6 Canada; Obstetrics and Gynecology, Princess Margaret Cancer Centre, University Health Network, Toronto, Ontario M5G 2M9 Canada; Daphne Cockwell School of Nursing, Ryerson University, Toronto, Ontario M5B 2K3 Canada

**Keywords:** Fear of cancer recurrence, Randomized clinical trial, Cognitive-existential group therapy, BREAST cancer, Gynecological cancer, Coping, Quality of life, Cancer distress

## Abstract

**Background:**

Clinically significant levels of fear of cancer recurrence (FCR) affect up to 49 % of cancer survivors and are more prevalent among women. FCR is associated with psychological distress, lower quality of life, and increased use of medical resources. Despite its prevalence, FCR is poorly addressed in clinical care. To address this problem, we first developed, and pilot tested a 6-week, 2 h, Cognitive-existential group intervention therapy that targeted FCR in survivors of breast or gynecological cancer. Following the positive outcome of the pilot, we are now testing this approach in a randomized clinical trial (RCT).

*Goal and hypotheses*: This multicenter, prospective RCT aims to test the efficacy of the intervention. The study hypotheses are that, compared to a control group, cancer survivors participating in the intervention (1) will have less FCR, (2) will show more favorable outcomes on the following measures: cancer-specific distress, quality of life, illness uncertainty, intolerance of uncertainty, perceived risk of cancer recurrence, and coping skills. We further postulate that the between-group differences will persist three and 6 months post-intervention.

**Methods:**

Sixteen groups of seven to nine women are being allocated to the intervention or the control group. The control group receives a 6-week, 2 h, structurally equivalent support group. We are recruiting 144 cancer survivors from four hospital sites in three Canadian cities. The sample size was based on the moderate pre/post-test changes found in our pilot study and adjusted to the drop-out rates. *Measurements*: The primary outcome, FCR, is measured by the Fear of Cancer Recurrence Inventory. Secondary outcomes measured include cancer-specific distress, perceived risk of cancer recurrence, illness uncertainty, intolerance of uncertainty, coping, and quality of life. We use reliable and recognized valid scales. Participants are to complete the questionnaire package at four times: before the first group session (baseline), immediately after the sixth session, and 3 and 6 months post-intervention. *Analysis*: In the descriptive analysis, comparison of group equivalent baseline variables, identification of confounding/intermediate variables and univariate analysis are planned. Each participant’s trajectory is calculated using Generalized Estimating Equation models to determine the time and group effects, after considering the correlation structures of the groups. An intent-to-treat analysis approach may be adopted.

**Discussion:**

Our Fear of Recurrence Therapy (FORT) intervention has direct implications for clinical service development to improve the quality of life for patients with breast (BC) and gynecological cancer (GC). Based on our pilot data, we are confident that the FORT intervention can guide the development of effective psychosocial cancer survivorship interventions to reduce FCR and improve psychological functioning among women with BC or GC.

**Trial registration:**

Dr. Christine Maheu registered the trial with ISRCTN registry (Registration number: ISRCTN83539618, date assigned 03/09/2014).

## Background

According to the 2015 Canadian Cancer Statistics, approximately 42 % of all women will be affected by cancer in their lifetime [1]. Although most cancer patients complete treatment and survive, survivors continue to struggle with fear of cancer recurrence [FCR]. FCR often describes a fear or a worry that cancer will return or progress in the same organ or into another part of the body [2, 3]. FCR is the most common problem reported by cancer survivors [3, 4], with reports ranging from 49 % in prostate cancer survivors to 74 % in lung cancer survivors [5]. A study involving a large cohort of long-term breast cancer survivors (*n* = 2671) found that fear of recurrence was highly prevalent (82 %) [6]. Despite the overrepresentation of women in this sample and that, gender’s association with FCR is still inconclusive although higher mean scores have been observed in women [7], the high prevalence rate underscores the need to address this concern in clinical practice.

While providing clinical care to such large group of cancer survivors may prove difficult, placing priority on individuals experiencing higher levels of FCR may be more feasible. From the literature, there is still no consensus on what constitutes low, moderate, and high levels of FCR. However, some current practice has been to classify low, moderate, and high based on the mean value of the total FCR scores used ± 1 SD [8]. In instances where a clinical cut-off of FCR has been established within a scale [9], this score tends to represent moderate to high levels. From the reported 2671 cohort reported in Koch et al. study [6], the majority had low levels of FCR with 17 % experiencing moderate to high levels. Compared to percentages found in systematic reviews, this percentage is relatively low. Systematic reviews of quantitative studies report prevalence rates of 49 to 66 % for moderate-to-high levels of FCR in cancer survivors [3, 8].

In a less recent cross-sectional study involving 1721 patients with mixed cancer sites and tumor stages, fear of disease progression or recurrence was found to be the most important single distress with 32.2 % of them rating this concern has strongly to very strongly distressed [5]. Their population was very heterogeneous, yet interesting determinants for psychological distress to prioritize for service delivery include patients with gynecological cancers within 6 months after diagnosis and cancer types with much longer treatment duration tending towards the 5-years survival rate criteria. Other systematic reviews demonstrate that high FCR measured at the end of treatment is a strong predictor of high long-term FCR [3, 8, 10, 11]. Overall, there is a trend for FCR to remain stable over the course of post cancer treatment [8].

Moderate-to-high levels of FCR are consistently associated with psychological distress, anxiety, depression, and stress-response symptoms (with correlations ranging from *r* = .19 to .69), suggesting relationships among these constructs, but not a strong overlap [3]. High levels of FCR are also associated with diminished physical and mental quality of life (QoL) [12–16], including increased uncertainty and worry about the future, difficulties making decisions about the future, and fear of death [13]. Cancer patients with high levels of FCR are more likely to refuse transfer from a specialized cancer center and to be followed-up care by a primary care provider [17]. They are less satisfied with their care [18], express doubt about whether their physicians are thorough enough [18], and are more likely to seek readmission to a cancer center [17]. Need for reassurance is often cited as the reason for increased frequency of hospital visits [19]. As such, cancer survivors with moderate-to-high levels of FCR tend to have maladaptive coping reactions, such as hypervigilance, excessive body checking and excessive need for reassurance seeking [20–23].

Despite clear evidence that cancer patients who exhibit moderate-to-high FCR have higher rates of psychological distress and may incur additional medical costs [24], few psychosocial interventions have been empirically tested to reduce FCR [3, 10, 11, 25]. This gap leaves clinicians ill-equipped to guide their patients with empirically tested interventions on how to reduce and cope with FCR. This gap prompted us to conduct a pilot study using a Cognitive-existential group approach. The results of our pilot study have been published [26, 27]. We are now testing the Cognitive-existential group While this approach is further being testing in a clinical trial named the Fear of Recurrence Therapy [FORT] intervention.

### Previous counseling approaches to fear of cancer recurrence

While there are several ongoing trials of FCR interventions [28–30], to date only one published intervention trial has addressed fear of disease progression, a concept related to FCR, in people with cancer or chronic arthritis [31]. This intervention involved both a cognitive behavioral group and a supportive-expressive group, and it successfully decreased fear of disease progression compared to a control group. However, all cancer participants were inpatients admitted to a rehabilitation clinic, and 20 % of them had recurrence or metastases. Hence, the concept of fear of disease progression addressed in this intervention may be more applicable to in-patients with active or advanced disease than to out-patients and those in remissions or with early-stage disease. Another intervention has been developed to address FCR among head-and-neck-cancer patients [32], but results of this trial have not yet been published.

Four additional studies evaluated group therapies designed to improve general psychological wellbeing outcomes for breast cancer survivors and reported on FCR as a secondary outcome. The first study evaluated the impact of a 6-session Mindfulness-Based Stress Reduction (MBSR) group and found a significant decrease in FCR immediately following the six sessions [33]. However, no follow-up study was conducted to evaluate the long-term effectiveness of the intervention, and the study did not include an attention only control group. The second study reported similar findings from an 8-session MBSR group [34] but, again, the study did not include an attention only control group, and the sample was small. The third study reported significant reductions in FCR immediately following a 12-week Emotion Regulation Group [35]. However, improvements in FCR were not sustained at 6 and 12 months after the intervention. Fourth, a telephone intervention designed to improve communication between breast cancer survivors and their physicians did not decrease FCR (the secondary outcome), although the intervention did improve the primary outcome, self-efficacy [36].

As described above, to date no intervention studies have reported an effective means of reducing FCR when it is the primary outcome for out-patient, disease-free survivors, with long-term impact and when the study includes an attention only control group. These gaps prompted us to develop the FORT intervention, a cognitive-existential (CE) group intervention that specifically targets FCR as its primary outcome and includes a structurally equivalent control group with post-intervention measurement at 6 months.

### Rationale for the cognitive-existential therapy intervention for fear of cancer recurrence

Cognitive-behavioural therapy (CBT) tends to be brief and structured, with a focus on skills in monitoring and modifying (in this case cancer-related and underlying fears) thoughts, emotions, and behaviors (e.g., building coping skills, relaxation training, goal setting, problem-solving) [37–39]. Most important for cancer survivors, CBT for health conditions now recognizes the importance of existential issues such as fear of the future and fear of death [37, 38]. Given the existential aspect associated with FCR, we decided to take a therapeutic approach that not only emphasized education, cognitive reappraisal, enhanced coping but also focused on existentially oriented strategies. Since many patients may not readily let go of their maladaptive coping reactions unless they can be helped to accept the ‘unspoken’ part of their experience (their fear of cancer recurrence), it was assessed that CBT alone would not likely help them.

To address patients’ fear, we chose the Cognitive-Existential (CE) approach elaborated by Kissane et al. [40, 41] to guide the development of FORT intervention, along with Lee-Jones et al.’s conceptual FCR model [42]. Themes in the CE approach relevant to our intervention include death anxiety, FCR, living with uncertainty, and future goals [41]. We also designed a group approach, rather than individual because group interventions are as efficacious in reducing distress among cancer patients as individual therapy [43, 44]. Group interventions offer several other advantages as well over individual therapy. In addition to cost-effectiveness, they allow participants to recognize their shared struggle and existential pain, to connect and learn from one another, and to feel understood and valued as they support one another [26]. In our pilot study of the group intervention, the women expressed appreciation in hearing other women’s strategies for dealing with FCR and shared inputs on how to improve the intervention [26]. The shared experiences facilitated group cohesion, making discussing sensitive topics, such as fear of treatment and death, more comfortable.

### Rationale for offering the intervention to breast and gynecological cancer survivors

The target populations for our RCT are breast and gynecological cancer (BC and GC) survivors. Although these two populations have different prognoses, they both have a high prevalence of moderate-to-high FCR [45, 46]. Furthermore, systematic reviews have found few if any differences in FCR scores by cancer type [3, 10, 25]. While testing interventions with more than one cancer type potentially increase the number of confounding factors to control for, it also increases the external validity of the intervention. Our RCT comprises the same two target populations as our pilot study. In the pilot study, analysis by cancer type revealed no group difference in the efficacy of the FORT intervention. We, therefore, expect that the two types of cancer survivors will respond similarly to the FORT intervention in the RCT. However, group assignments to the intervention are by cancer type.

### Aims of RCT

The aim of this RCT is to test the effectiveness of a cognitive-existential (CE) group intervention, the FORT intervention, in reducing fear of cancer recurrence among breast (BC) or gynecological (GC) cancer survivors.

### Research question and hypotheses

The research questions are as follow:

What is the effectiveness of the FORT intervention (2 h group sessions for 6 weeks), compared with a structurally equivalent control group, in reducing FCR in the short term (immediately after the end of the intervention at T2) and in the long term, at 3 months (T3) and 6 months (T4) after the intervention?

The main hypotheses are as follow:

1. Compared to a structurally equivalent control group, BC and GC survivors participating in the FORT intervention will (a) have less FCR and (b) show greater improvement in the following secondary outcomes: cancer-specific distress, coping skills, illness uncertainty, quality of life, intolerance of uncertainty, and perceived risk of cancer recurrence.
2. Group differences will be maintained over the 6 months following the intervention, at T3 (3 months post-intervention) and T4 (6 months post-intervention).

## Methods/design

We are conducting this RCT with four specialized cancer centers in two Canadian provinces. The study is funded by the Canadian Cancer Society and is registered with the ISRCTN under Dr. Maheu (Clinical Trial No. ISRCTN83539618, date assigned 03/09/2014). Ethical approval has been obtained from all four participating centers. All possible precautions were taken to safeguard the rights and privacy of the participants.

### Participant selection

Patients are eligible to participate if they:

- Have a confirmed past diagnosis of breast (BC) or gynecological cancer (GC), in stages 0–3;
- Are disease-free at the start of the group;
- Are 18 years or older;
- Have completed treatment, except for targeted therapy or hormonal therapy;
- Have a score of 13 or greater on the severity subscale of the Fear of Cancer Recurrence Inventory (range 0–36), suggesting clinical levels of fear of cancer recurrence (FCR) [9];
- Have a score of at least 24 on the cancer-specific distress measure of the Impact of Events Scale (range 0–75), indicating clinical levels of distress [47, 48].
- Can read and write English; and
- Can give informed consent.

If participants develop a recurrence in the course of the study, they remain in the group intervention, but their follow-up data are not used in the study.

Patients are ineligible to participate if they:

- Had a previous cancer recurrence;
- Are enrolled in another psychotherapy group at the start of the study or during their six sessions;
- Have an unresolved mental health disorder based on disclosure by the potential participant that may affect the group’s work as assessed by the group leader at the pre-interview session.

### Participant recruitment

We are recruiting 144 BC or GC survivors from four hospital sites: Princess Margaret Hospital and Mount Sinai Hospital in Toronto, Ontario, the Ottawa Hospital in Ottawa, Ontario, and the Jewish General Hospital in Montreal, Quebec. Both the Toronto and Ottawa sites see approximately 300 BC and 250 GC patients a year. The Toronto sites are providing 4 to 5 iterations of the FORT intervention; the Ottawa site is providing 3; and the Montreal site one. Because of the higher number of BC patients than GC patients served by these centers, 75 % of the groups will comprise BC patients. Each group (the FORT and the structurally equivalent group control) comprises 6 to 8 women, but up to 9 women per group are being recruited to accommodate possible drop-outs.

Each recruiting site has placed recruiting posters in its waiting area, as well as beside key elevators that patients use on follow-up visits. We also work with the sites’ medical staff to inform eligible participants about the study and seek their consent to be contacted by a research assistant (RA). When the RA contacts potential participants, she explains the study and screens them for eligibility. Study pamphlets with tear-off forms in which potential participants can give their contact information are also placed in the waiting areas of BC and GC follow-up clinics. A locked drop box is left at each participating sites waiting areas for potential participants to insert their contact information.

Following confirmation of eligibility, patients are scheduled to meet with one of the group leaders for a pre-interview assessment. The goal is to review group-work expectations and to assess whether group work fits with patients’ expectations. If both parties agree that group work is a good match, study information is reviewed, written consent is obtained, and a copy of the baseline assessment questionnaire is given to the patient, along with a stamped, pre-addressed envelope. Potential participants are advised to mail in their baseline assessment questionnaires before the start of the first group session.

### Randomization of participants

Before randomization, all of the following forms are completed by all eligible and consenting participants: (a) eligibility form, (b) consent form, (c) baseline measures, and (d) baseline interview form. Randomization of all recruiting sites is centralized at one recruiting site and is performed by a biostatistician working at arm’s length from the study. The biostatistician does not know the identity of the therapists and gives the group allocations to the study’s RA. The group allocation is concealed to the participants. The participants do not know which groups comprise the FORT intervention or the structurally equivalent control group. Randomization occurs each time 18 BC survivors or 18 GC survivors are deemed eligible at one of the four sites. This ratio of 18 ensures nine women in each group, with a buffer of at least two probable dropouts per group. We achieved this target in our pilot study. Each group is planned to start within 2 weeks of the initial randomization of women to each study arm.

### The fear of recurrence therapy intervention

The FORT intervention is theoretically guided by the FCRM [26, 27], Leventhal’s Common Sense Model [42, 49], Mishel’s Uncertainty in Illness Theory [50], and cognitive model of worry [51].

#### Perceived risk of recurrence

According to Leventhal’s Common Sense Model, FCR is best viewed as a multidimensional construct in which internal and external cues increase perceived risk of recurrence, which in turn heightens FCR [15, 42, 52]. Examples of internal triggers include aches and pains. External triggers include anniversary dates of cancer diagnosis, attending cancer screening appointments, and hearing of a friend with a recurrence. There is evidence that perceived risk may be the link between triggers and FCR, as suggested by Leventhal’s Common Sense Model [42]. Perceived risk of recurrence tends to lead survivors to focus on sensations that, before their cancer diagnosis, would have been viewed as normal or, at least, benign (e.g., occasional pain) and to now interpret these as evidence of recurrence. To influence levels of perceived risk, in the intervention, participants are taught to identify internal and external triggers and their link with FCR.

#### Coping strategies

Once patients perceive a risk of recurrence, there is increased the likelihood of maladaptive coping and behavior such as anxious preoccupation, excessive body checking, and reassurance seeking, including from health practitioners [32]. Strong empirical evidence supports the reciprocal relationship between coping and FCR. Patients with moderate-to-high FCR become preoccupied and report excessive body checking [53], reassurance seeking [3], and avoidance coping such as pushing away intrusive thoughts of possible recurrence [54, 55]. While such coping strategies provide temporary relief at best, they tend to increase FCR over time by feeding hyper-vigilance and reinforcing maladaptive coping behaviors [19, 32, 42]. Our FORT intervention focuses on identifying maladaptive coping behaviors and their consequences and then replacing these with more favorable coping skills.

#### Uncertainty

According to Uncertainty in Illness Theory [50], uncertainty is generated when components of an illness possess the characteristics of inconsistency, randomness, complexity, unpredictability, and lack of information in situations of importance to the individual [56]. Living with constant uncertainty increases psychological distress and perceived risk, and reduces the quality of life [50, 56]. To reduce uncertainty about living with cancer, participants learn about the signs that indicate actual cancer recurrence vs. benign symptoms.

#### Intolerance of uncertainty and faulty beliefs about the benefits of worrying

Cognitive models of worry suggest that worriers have a lower tolerance for uncertainty than non-worriers [51]. Patients with elevated FCR may consider inadequate anything less than the complete certainty that they are cancer-free, which may explain their increased use of coping strategies, such as seeking medical reassurance or body checking. While uncertainty indicates ambiguity associated with cancer and its treatment, intolerance of uncertainty reflects a difficulty in tolerating and coping with even small amounts of ambiguity or uncertainty in facing cancer. Cognitive models of worry also suggest that worriers tend to believe that worrying is beneficial. By worrying, they think they can prevent negative outcomes. In our FORT intervention, participants are taught to challenge their beliefs in the benefits of worry and to develop new coping skills to tolerate better uncertainty.

#### Content and processes of the intervention

Two leaders facilitate each group (for both the intervention and control groups). Table 1 gives the content of the FORT group sessions. Group leaders cannot add new participants once a six-session group has started. This procedure is necessary to enhance group cohesiveness and consistency [57]. At their pre-study-interview sessions, participants are told that, if they are absent for more than one group session, they will be asked to withdraw from the study, because too much material and group process development will have been missed. However, the participants are told that, if they have to be absent for only one session, one of their group leaders will contact them to review the session’s content and the homework to be done before the next session. When participants miss more than one session, they are withdrawn from the group and then invited to join a group that has not yet started. Traveling expenses of both the FORT intervention and the control groups are reimbursed. Both groups are provided with refreshments 30 min before the start of each weekly session to allow group members to settle in and chitchat. This strategy increases group cohesion and facilitates starting the group on time.

**Table 1:** Content of the Fear of Recurrence Therapy (FORT) intervention sessions

Session number	Description
1.	- Self-introduction by each participant, with a focus on their experience with fear of cancer recurrence.
	- Introduce major heading of the Fear of Recurrence Therapy (FORT) from the six sessions.
	- Introduce the automatic thought, behavior, and consequence therapy model and the fear of cancer recurrence model (FCRM).
	- Introduce notions of cognitive restructuring and how to identify FCR triggers.
	- Teach and practice progressive muscle relaxation.
	Homework: Practice progressive muscle relaxation daily. Complete thought journal and challenge maladaptive thinking about the fear of cancer recurrence.
2.	- Prepare questions for nurse specialist, who provides education about general cancer screening guidelines and signs of recurrence in Session 3.
	- Help participants deal with the fact that uncertainty can never be eliminated.
	- Discuss ways of regaining a sense of control using Wheel of Life exercise.
	- Teach calming self-talk. Provide participants with relaxation CD audio and instruct them to use calming self-talk phrases with their relaxation CD when appropriate.
	Homework: Listen to CD every day. Practice calming self-talk. Complete thought journal and challenge maladaptive thinking about the fear of cancer recurrence. Prepare questions for the specialist nurse visit in Session 3.
3.	- Visit from nurse specialist.
	- Increase tolerance for uncertainty by discussing acceptable levels of worry.
	- Challenge faulty beliefs about benefits of worry.
	- Decrease maladaptive coping strategies.
	- Teach and practice guided imagery.
	Homework: Practice guided imagery daily. Continue challenging faulty beliefs about benefits of worry. Complete thought journal, adding a column for behaviors to monitor coping strategies that participants are adopting.
4.	- Provide psycho-education about worry and the need for exposure to worst fears.
	- Promote emotion expression and confront specific fears that underlie fear of cancer recurrence.
	- Write down worst-fear scenario.
	- Teach and practice mindfulness using a body scan through breathing exercise.
	Homework: Read worst-fear scenario every day and then do a self-care activity. Practice mindfulness exercises daily.
5.	- Review exposure to worst fear scenario exercise.
	- Discuss ways of coping with some of the feared outcomes.
	- Promote expression of demoralization feelings.
	- Encourage participants to re-engage with important life goals, people or activities they may have given up.
	- Discuss what meanings the future and planning now have for them.
	- Teach and practice mindfulness using eating meditation (Raisin meditation).
	Homework: Challenge their worst fear scenarios into more likely realistic ones. Write down goals and priorities for the future.
6.	- Review all content covered.
	- Discuss future goals.
	- Set new priorities.
	- Promote saying good-bye to the group and provide closure.

At the first session, participants in the FORT intervention receive a binder describing the intervention’s framework as well as each session’s activities. At the beginning of the first session, group leaders review the key goals of the intervention. *The key goals of the FORT intervention are to:*

1. Distinguish worrisome symptoms from benign ones.
2. Identify FCR triggers and inappropriate coping strategies.
3. Facilitate the learning and use of new coping strategies, such as relaxation techniques and cognitive restructuring.
4. Increase tolerance for uncertainty.
5. Promote emotional expression of specific fears that underlie fear of cancer recurrence.
6. Reexamine life priorities and set realistic goals for the future.

The participants are shown the in-group activities and the short assignments to complete at home. The latter reinforce concepts learned during the group sessions and help participants prepare for the next session’s content. At the end of each session, take-home homework’s for the following week is reviewed and, at the beginning of the next session, participants’ homework’s is reviewed. As part of their study package, participants receive a CD or audio file containing six different relaxation techniques (deep breathing, progressive muscular relaxation, guided imagery, body scanning, deep relaxation, and encouraging sleep). Each of these techniques is discussed or practiced in one of the group sessions.

#### Content of the control group intervention

Participants assigned to the control group receive a structurally equivalent intervention to rule out the possibility that attention received from FORT-group facilitators and the social support received during the FORT-group sessions, rather than the FORT intervention itself, is responsible for the FORT intervention’s effectiveness. To this end, the control group sessions have a structure similar to the FORT but different content. Specifically, the control group receives six, weekly, 2 h sessions, delivered by two health care professionals. Although the sessions focus on the challenges of living with a cancer diagnosis, the six sessions comprise a general support group but do not include the unique ingredients of the FORT intervention. Nonetheless, the intervention and control groups possess common factors in equal measure such as length and number of sessions, and both led by two health professionals [58]. During the control group sessions, the following topics are discussed: (a) exploring what it means when you find out you have cancer, (b) coping with post-treatment symptoms and side effects, (c) challenges of living with a cancer diagnosis, (d) incorporating wellness into your daily life, (e) building a survivorship plan, and (f) managing transitions (from active treatment to resuming work and family life). While the group leaders are instructed to create a supportive atmosphere in which patients may share their worries about living with a cancer diagnosis, the therapists are instructed not to teach the linkages between emotions and behaviors. Specifically, in the control group, potential therapeutic ingredients found in the FORT intervention group, such as cognitive reframing, building coping skills, or exposure to participants’ worst fears are not introduced. Group leaders in the control group are instructed to use reflection and restatement when participants do introduce these topics DL Safer and EM Hugo [58]. Although the control group comprises specific weekly topics, the content, and flow of each session are not highly scripted, no homework is assigned, and no relaxation exercises are taught during the sessions or practiced at home. The group leaders are expected to have clinical experience with cancer patients and general knowledge about the weekly topics.

#### Standardization and training in the FORT intervention and control intervention

To set performance standards and ensure treatment fidelity [59–61], we developed a FORT intervention manual and tested it in the pilot study for dosage (number of sessions) and intensity (duration of each session). After this pre-testing, the manual was refined for the RCT. All FORT facilitators receive the same 2-day training, under the direction of the first, second, third, and fourth authors, all of whom are well trained in Cognitive existential therapy. FORT leaders also attend annual, 1-day training refreshers to maintain their competency and to avoid therapist drift-off (e.g., expanding the session content based on insights from their clinical experience). We used this approach to monitoring treatment integrity and fidelity successfully in our pilot study.

Two specific control group leaders, with previous experience in delivering support groups to women with cancer, were asked by the research team to develop a manual for group leaders and participants, based on six established topics for the control group. The two control-group leaders did not participate in the FORT intervention training, to reduce risk of content contamination, and they provided training to group leaders at the other intervention sites. Analogous quality-control fidelity checks for reliability and consistency are done on both intervention and control groups.

All group sessions are audio-recorded and reviewed, with participants’ permission. The audio-recording are accessible only to the research team. The first and second authors independently and randomly review 20 % of all recordings, using fidelity checklists built from each of the intervention manuals. The checklists have two ratings: (a) areas covered (yes or no) and (b) quantity and quality of area covered (0–2). The second rating assesses adherence to the manuals, adequate processing of affect in the FORT intervention, and efficient time management. When adherence to the manuals is less than 80 %, the first and second authors review with group leaders how to improve adherence.

### Pilot study of the intervention

The pilot study of the FORT intervention was carried out between October 2010 and October 2012, with funding from the Canadian Institutes of Health Research [26, 27]. The pilot study assessed the feasibility and preliminary efficacy of the FORT intervention for 56 participants with either BC or GC. Briefly, we found that the intervention was feasible and showed promising efficacy. Specifically, women who took part in the intervention reported significant reductions in the primary outcome, FCR, and the secondary outcomes uncertainty, coping, cancer-specific distress, and quality of life, as measured before and immediately after the intervention. These changes were maintained 3 months after the intervention ended. Our RCT builds on the findings of the pilot study by comparing the FORT intervention to a structurally equivalent control group. In planning the RCT, we carefully took into account the issues found in the pilot study. First, although most psychological measures showed improved outcomes, we observed changes on only four of the 14 coping subscales, This result may suggest that the coping scale we chose for the pilot study may not have been specific enough to measure changes in other coping behaviours such as for cognitive avoidance, reassurance seeking, body checking that we had aimed to reduce, as based on our theoretical FCRM [27]. For the RCT, we, therefore, revised our coping measures to align them more closely with our theoretical model. Second, in the pilot study, we used an FCR measure that did not have a validated cut-off score for identifying clinically significant levels of FCR. Recent developments in psychometrics have made it possible to distinguish survivors with minimal FCR from those with clinical levels of FCR [11]. Consequently, for the RCT, we changed our measurement of FCR scale to a more reliable scale, the Fear of Cancer Recurrence Inventory, which has a validated clinical cut-off score based one of its subscales, the FCR severity scale [9]. The scale also provides a total FCR score [9]. We also reorganized the content of the weekly sessions, based on participants’ feedback [26]. One example was moving the nurse specialist’s presentation on cancer screening follow-ups from Session 2 to Session 3, to allow participants to learn coping skills for confronting anxiety associated with hearing about cancer risk. Finally, in the pilot study whereas therapeutic gains were not assessed beyond 3 months, the RCT includes a 6-month follow-up measurement.

### Primary and secondary outcomes in the RCT

The theoretical model for our pilot study guided the choice of measures included in the RCT questionnaire package. There are four measurement time-points for participants randomized to either the intervention or the control group: 2 weeks before the intervention (T1) 1 week after the intervention (T2), and 3 and 6 months after the intervention ends (T3 and T4, respectively). The first questionnaire package (T1) includes additional measures to evaluate the intervention and control groups’ comparability, such as demographic data including age and socioeconomic status, and medical history of cancer diagnosis.

The primary outcome of the study, *fear of cancer recurrence*, is measured by the Fear of Cancer Recurrence Inventory (FCRI) [9]. This 42-item questionnaire includes a total score as well as seven subscales, measuring triggers, FCR severity, psychological distress, functional impairment, insight, reassurance, and coping strategies. A score of 13 or higher on the nine-item severity subscale (range 0–3) indicates a clinical level of FCR [62]. The FCRI has been shown to have adequate reliability and validity (construct validity *r* = 0.68 to 0.77; reliability scores α = 0.95) [9].

The secondary outcomes are measured as follows: *Cancer-specific distress* is measured by the Impact of Event Scale (IES) [47], a 15-item questionnaire that assesses cancer distress. The IES has two subscales: intrusive thoughts and avoidance. The scale has good internal consistency (α = 0.84–0.91) and satisfactory test-retest reliability (total *r* = 0.80) [63]. *Perceived risk of cancer recurrence* is measured by a single-item question asking respondents to indicate their level of perceived personal risk for cancer recurrence over the last two days [64]. *Intolerance of uncertainty* is measured by the Intolerance of Uncertainty Scale (IUS) [65]. The IUS is a 27-item, four-factor questionnaire that presents uncertainty as stressful and upsetting, uncertainty as leading to the inability to act, uncertain events as being negative and to be avoided, and being uncertain as unfair [65]. The IUS has a reliability coefficient of *r* = 0.74 [65]. *Uncertainty in Illness* is measured by the Mishel Uncertainty in Illness Scale–Community version [56], which consists of 23 items rated on a five-point Likert scale. For cancer, this scale has alpha coefficients of 0.90 [66]. *Coping* is measured by the following three coping scales: (a) *Cognitive Avoidance Questionnaire* [67], a measure of avoidance coping of 25 items, (b) the *Reassurance-Seeking Behaviours subscale* of the Health Anxiety Questionnaire [68], a measure of body-checking and seeking reassurance of 3 items, and (c) the *Reassurance Questionnaire* [69], a measure of seeking reassurance from physicians of 10 items These coping measures have been extensively used for cancer patients as well as with other patients who have health anxiety. The measures have demonstrated satisfactory reliability and validity [56, 67, 70, 71]. *The quality of life* is measured by the SF-8 instrument [72], a health-related quality of life measure that assesses general physical and mental health within a 4-week recall period. Cronbach’s alpha internal consistency for both subscales is 0.61-0.68 [73].

#### Potential covariates

As group cohesion may influence the intervention’s impact, we measure group cohesion at the end of each 6-week FORT-intervention group and each 6-week control group intervention. Cohesion is measured using the *Group Cohesion Scale − Revised* [74], a 25-item scale measuring interaction and communication among group members. For example, one item is “Group members usually feel free to share information.” The Cronbach’s alpha internal consistency of this scale ranged from .77 to .90 during post-test assessment [74].

Group alliance within therapy groups can be therapeutic in and of itself and can enhance intervention efficacy [75]. That is, patients who perceive improvements during their therapy are more likely to have positive feelings towards the therapist, be more committed to treatment, and work more collaboratively. We measure group alliance using the *California Psychotherapy Alliance Scale* [76], a 24-item scale with four theoretically derived dimensions: (a) the therapeutic alliance, (b) the working alliance, (c) the therapist’s contribution to the alliance, and (d) the agreement on goals and tasks of therapy. We measure therapeutic-working-group alliance immediately following the last sessions of both the intervention and the control groups.

Treatment credibility and participants’ expectancy for improvement are two additional variables that may contribute to alternative explanations of differences found between two compared conditions [77]. To assess credibility and expectancy to ensure initial equivalency between the FORT intervention and the structurally equivalent control group, we are using the *Credibility/Expectancy Questionnaire* (CEQ) [77]. The CEQ has 6 items; three items relate to the credibility factor (“think” questions), and three items relate to the expectancy factor (“feel” questions). Each factor demonstrates high internal consistency (α = 0.81 for expectancy and α = 0.86 for credibility) and a good Cronbach’s alpha for the whole scale (α = 0.85) [77]. Test-retest reliability over a 1-week period scored at 0.82 for expectancy and 0.75 for credibility [77]. The CEQ has two ratings; one is from 1 (not at all) to 9 (very much), and the other from 0 % (not at all) to 100 % (very much). Scoring involves transforming the percentage to a number between 1 and 9, giving both scales a range from 3 to 27. The CEQ is given to participants immediately after the end of Session 1, in which the study intervention is explained. Both the FORT intervention and the control group members complete this scale.

#### Sample size

The primary study outcome is FCR, as measured by the FCRI [9]. The sample size was calculated based on the moderate changes in FCR we observed in the pilot study from baseline (T1) to 3 months post intervention [27] and in the only published RCT intervention study that addresses the fear of disease progression [78]. A sample size of at least 112 participants ensures our ability to detect an effect size of 0.40, with a power of 0.95 and an alpha level of .05 between pre- and post-intervention FCR measures. Therefore, we plan to recruit eight groups of seven participants, for a total of 56 participants per arm. We are also enrolling an additional two participants per group per arm, to account for possible dropouts (72 per arm). This strategy will yield 144 participants in total.

#### Statistical analysis

Random allocation should help us control for possible extraneous variations. However, we are also measuring and controlling for known extraneous variables (if needed) that could influence our primary outcome. Age, education, income, and cancer stage have been identified as possible predictors of FCR [3, 10]. These are being considered as potential covariates and are measured at baseline (T1).

Descriptive statistics are being used to present the study participants’ characteristics. To determine the effectiveness of our intervention, we are using advanced regression analysis. Kaplan-Meier curves, period prevalence, and cumulative risk models may be used to explain the time-event analysis. To analyse data from across the four-time points (T1-T4), we are using Generalized Estimating Equations (GEE) models, with factor-specific events for each of the outcome variables. Similar sensitivity analyses are to be used to assess the effect of the FORT intervention on the secondary outcomes. All models will be adjusted for adverse events and other potential confounders. We will also assess treatment benefits for subgroups of participants by analyzing their sociodemographic, clinical, and psychological characteristics [79]. To identify key variables that lead to changes in our outcome variables, we are taking two approaches to selecting the variables for our regression analyses, including intermediate variables. In the first approach, variables will be selected based on background knowledge and the relationship of the variable to intervention and outcomes. Our second approach relies primarily on high-dimensional, automatic variable selection techniques, such as forward and backward selection, and automatic high-dimensional ‘Proxy’ adjustment [80]. In our opinion, combining these two approaches yields more unbiased estimates. For the above analyses, we will use the statistical software packages IBM SPSS Statistics 22.0 and STATA 14.

## Discussion

### Clinical significance and contributions

Our Fear of Recurrence Therapy (FORT) intervention has direct implications for clinical service development to improve the quality of life for patients with breast and gynecological cancer. Based on our pilot data, we are confident that the FORT intervention can guide the development of effective psychosocial cancer survivorship interventions to reduce FCR and improve psychological functioning among women with BC or GC. Having a FORT manual developed, psychosocial oncology professionals can be easily trained to provide FORT to cancer patients who voice concerns about FCR. The therapy is also highly adaptable to other non-group formats, such as individual, in-person, telephone counseling, or telehealth. Studies are currently underway testing the FORT intervention in individual [81] and telehealth formats. It can also be adapted for patients with other cancer types.

Currently, there is a lack of consensus on the definition, measurement, and theoretical formulation of FCR. Our RCT findings will contribute to a better understanding of FCR predictors and the cognitive and emotional interplay involved in the formulation of FCR. Finally, the RCT will contribute to improving cancer survivorship programs and development of clinical guidelines for addressing fear of cancer recurrence.

## Abbreviations

BC: breast cancer
CBT: cognitive behavioural therapy
CE: cognitive-existential
FCR: fear of cancer recurrence
FCRI: fear of cancer recurrence inventory
FORT: fear of recurrence therapy
GC: gynecological cancer
GEE: generalized estimating equations
IES: impact of event scale
IUS: intolerance of uncertainty scale
MBSR: mindfulness-based stress reduction
QoL: quality of life
RCT: randomized controlled trial
